# Editorial: Mathematical modeling and computational predictions in oncoimmunology

**DOI:** 10.3389/fimmu.2024.1432372

**Published:** 2024-05-30

**Authors:** Vladimir A. Kuznetsov, Heiko Enderling, Mark Chaplain

**Affiliations:** ^1^Urology, Upstate Medical University, Syracuse, NY, United States; ^2^Biochemistry and Molecular Biology, Upstate Medical University, Syracuse, NY, United States; ^3^Department of Radiation Oncology, University of Texas MD Anderson Cancer Center, Houston, TX, United States; ^4^School of Mathematics and Statistics, University of St Andrews, St Andrews, United Kingdom

**Keywords:** oncoimmunology, mathematical modeling, nonlinear dynamics, computational prediction, immune response, immunotherapy

The immune system can recognize and kill malignant cells. Anti-cancer immune mechanisms are realized as multiscale, nonlinear cellular and molecular processes. Many factors determine the outcome of immune system-tumor interactions, including cancer-associated antigens, immune cells, and host organisms. In the context of such complexity and non-linear dynamics, deep data-driven theory and mathematical modeling enables an increase in our understanding of mechanisms controlling these processes, defining reliable biomarkers, and potentially improving the capability of immune and combined therapies.

Here we review and summarize the contributions to the Research Topic “*Mathematical modeling and computational predictions in oncoimmunology*.”

The review by Metzcar et al. deals with the overview of a mechanistic learning approach that combines mechanistic mathematical modeling with data-driven machine learning. The authors reviewed the perspective of this approach and discussed how mechanistic learning may advance mathematical oncology. The four categories of mechanistic learning (sequential, parallel, extrinsic, intrinsic) are presented with examples from oncology research such as longitudinal tumor response predictions and time-to-event analyses.

Boklund et al. develop a mathematical model of myeloproliferative neoplasm (MPN) progression and treatment of MPN with JAK1/2 inhibitor ruxolitinib. A system of ordinary differential equations (ODUs) describes and models the dynamics of the interaction between healthy cells, malignant cells, and inflammatory cytokines produced by the immune system. By fitting the model to data, the authors show that the proposed mechanisms of ruxolitinib action are compatible with the disease dynamics. The model analysis and data fitting enable computational predictions and uncertainty quantification of the future course of the disease for individual patients based on longitudinal measurements of the MPN load.

In their article, Ledzewicz and Schättler develop a model of the tumor treatment effects when traditional chemo- and radiotherapies are combined with immunotherapy. Based on the classical Stepanova (1980) and Kuznetsov (1994) papers, a dynamical system model of the low-dimensional cellular immune response against cancer antigen(s) is formulated. It is shown that the system’s dynamics is dependent upon the values of its parameters, and encompasses the 3E’s (elimination, equilibrium, escape) of tumor antigen(s)-immune system interactions. Using numerical analysis, optimal control strategies for implementing immunotherapy, chemotherapy, and radiotherapy are formulated to reverse the tumor ‘escape’ scenario.

Castorina et al. analyze the Gompertz population growth law to evaluate radiation and immunotherapy effects on tumor mass dynamics in individual patients. Using time course data, they show that the Gompertz law can describe therapy effects with two effective parameters. Their macroscopic approach quantifies the treatment/interaction effect between the immune system, radiotherapy, and tumor progression. This result permits quantitative tumor mass kinetics data analyses, which give useful indications for disease progression and assess the potential efficacy of radiotherapy and immunotherapy combinations.

Adhikarla et al. validate the previously reported mathematical model against experimental data where the timing of the CAR-T cell therapy is varied keeping the targeted alpha particle therapy (TAT) dosage and timing constant. Model parameters are elucidated from the experimental data to optimize the timing of CAR-T cell therapy post-TAT by maximizing progression-free survival. The effect of fractionated dosing of both TRT and CAR-T cell therapies on the survival metrics is also studied. In addition, multiple dosing strategies for TRT and CAR-T cell therapies are tested to analyze whether the splitting and scheduling of the doses result in improved tumor control or survival.

To assess the combined effects of anti-PD-1 and anti-FGFR3 small molecule inhibitors (SMI) on tumor growth and the immune response, Bergman et al. build agent-based models (ABM) that capture key aspects of tumor heterogeneity and CD8+ T cell phenotypes, their spatial interactions, and their response to therapeutics. The model quantifies how tumor antigenicity and FGFR3 activating mutations impact the disease process and response to anti-PD-1 antibodies and anti-FGFR3 SMI. The modeling results indicate the need to quantify FGFR3 signaling, and the fitness advantage conferred on bladder cancer cells harboring this mutation.

Yosef and Bunimovich-Medrazitsky present a theoretical investigation of non-muscle invasive bladder cancer (NMIBC) growth and its continuous treatment with Mitomycin-C (MMC). Based on biological data analysis and using ordinary differential equations (ODEs) the authors describe the cells and drug molecule dynamics of tumor-immune cell interactions during MMC treatment. Several hypothetical scenarios for NMIBC depict the evolution of tumors for small, medium, and large tumors. The results offer the possibility to improve MMC chemotherapy for NMIBC patients and explain some of the mysteries of NMIBC chemotherapy.

West et al. present a multiscale mathematical model that captures the phenotypic, vascular, microenvironmental, and spatial heterogeneity that shapes acid-mediated invasion and immune escape over a biologically realistic time scale. The model explores several immune escape mechanisms such as i) acid inactivation of immune cells, ii) competition for glucose, and iii) inhibitory immune checkpoint receptor expression (PD-L1). This model helps to decompose the key constraints on evolutionary costs and benefits of three key phenotypic axes on tumor invasion and treatment: acid resistance, glycolysis, and PD-L1 expression. The benefits of concomitant anti-PD-L1 and buffer treatments are promising treatments to limit the adverse effects of immune escape.

Lems et al. extend an existing mathematical model and embed it in multi-scale spatial simulations to describe the effects of immunosuppression and spatial heterogeneity on the crosstalk between EMT and IFNγ-induced PD-L1 expression. This analysis demonstrates that the relation between PD-L1 expression and EMT status is highly complex, and depends on the forms of immunosuppression established by the tumor as well as on spatial heterogeneity concerning cytokines influencing these pathways. These results may contribute to the development of therapeutic strategies for effectively combating metastatic dissemination, as well as immune evasion.

## Conclusion

The collected articles demonstrate that mathematical modeling and computational simulation contribute to oncoimmunology by consolidating experimental, theoretical, and clinical facts into quantitatively/qualitatively predictive and hypothesis-driven frameworks. Moreover, the modeling allows the prediction and validation of specific mechanisms and key parameters of the studied complex systems *in vitro* and *in vivo*. By computational analysis of independent data identifying molecular and cellular mechanisms and interaction parameters, mathematical models can be useful tools to stratify immunotherapy responses and personalize outcomes for cancer patients.

## Author contributions

VK: Writing – review & editing, Writing – original draft. HE: Writing – review & editing, Writing – original draft. MC: Writing – review & editing, Writing – original draft.

